# Atomic-scale understanding of the Na and Cl trapping on the Mo_1.33_C(OH)_2_-MXene

**DOI:** 10.1038/s41598-022-12177-6

**Published:** 2022-05-18

**Authors:** J. Guerrero-Sanchez, Dalia M. Muñoz-Pizza, Ma Guadalupe Moreno-Armenta, Noboru Takeuchi

**Affiliations:** 1 Centro de Nanociencias y Nanotecnología, Universidad Nacional Autónoma de México, km.107, Apdo. Postal 14. Carretera Tijuana-Ensenada, Ensenada, Baja California México; 2 Departamento de Estudios Urbanos y del Medio Ambiente, Colegio de la Frontera Norte, Tijuana, Baja California Mexico; 3 Facultad de Ciencias Marinas, Universidad Autónoma de Baja California, Ensenada, Baja California Mexico

**Keywords:** Two-dimensional materials, Two-dimensional materials

## Abstract

Drinking water scarcity in arid and semi-arid regions is a reality that may turn into a global healthcare problem in the next few years. The scientific community is always looking for new materials to achieve effective sea and brackish water desalination to reduce water scarcity. Commonly, theoretical, and experimental methods make a synergy to better understand and explain the chemical and physical processes in water desalination electrodes. In this way, experimental evidence pointed Mo_1.33_CT_x_ MXene as an efficient ion intercalation material, in which both Na^+^ and Cl^−^ are removed. However, the atomic scale understanding of the physicochemical processes due to the Na and Cl interaction with the MXene is still unknown. We report the Na^0^ and Cl^0^ interaction with an OH functionalized Mo_1.33_C monolayer through a comprehensive first-principles density functional theory assessment. Results demonstrate that Na atoms attach to Oxygen, whereas Cl atoms bond through hydrogen bonds to the functional groups in the MXene, these bonds have two energy contributions: electrostatic and charge transfer, which increases its adsorption energy. Electrostatic potential isosurfaces, Bader charge analysis, and non-covalent interactions index help clarifying the way Na^0^ and Cl^0^ attach to the MXene layer. Oxygen atoms have an affinity for the electropositive Na^0^ atoms, which after interaction oxidizes to Na^+^, whereas hydrogen atoms—of the hydroxyl groups—interact with the electronegative Cl^0^ atoms, which upon adsorption reduce to Cl^−^. Our findings explain why OH-functionalized Mo_1.33_C can efficiently remove both Na and Cl atoms based on their affinities with the functional groups present in the MXene layer.

## Introduction

Freshwater scarcity is a critical global problem. Global warming and the increased demand for agricultural, domestic, and industrial use are drivers of the lower availability of this resource. Additionally, limitations in the development of circular systems and pollution^1^ generate freshwater scarcity as well. Between the alternatives to obtain clean water, the desalination process is widely considered. Despite the growing number of desalination plants, currently 17,000^2^, there are important challenges due to their high costs of operation^3^, mainly in energy consumption. The desalination technologies include membrane distillation, electrodialysis, reverse osmosis, ion exchange, thermal based processes, and nanofiltration^4,5^.

Nanofiltration is applied to improve the performance in the desalination technologies, allowing operation at low pressures, energy efficiency, and increase in the permeability and selectivity^6^. Application to improve salt rejection, ion exchange and hydrophobic characteristics have been proved using carbon nanotubes^7,8^, TiO_2_ nanofilms^9^, and graphene oxide nanotubes^10^. Recently the use of the two-dimensional MXenes family has been recognized with potential applications on seawater desalination due to the high capacity of rejection, permeability, and selectivity^11,12^.

2D MXenes were discovered in 2011 by Michael Naguib et al.^13^. They reported a new 2D material—Ti_3_C_2_T_x_—with a potential wide range of applications. This paper established the birth of a new family of versatile 2D materials, MXenes, with applications that span from energy, optical, electronic, environmental, sensors, catalysis, and several other^14^. Mono-metal MXenes could be achieved upon exfoliating them from their 3D counterpart, the MAX phases. These have the general formula M_n+1_X_n_T_x_, (n = 1, 2, 3). M stands for an early transition metal, X = C or N, and T_x_ represents the functional groups attached to the surface (O, OH, and F)^15^. Until today, just some Mono-metal MXenes have been fabricated in the lab. However, it is expected to have a large quantity of these materials since there exist > 70 MAX phases^16^. Mono-metal MXenes—carbides or nitrides—have seven, five, and three layers, in which a transition metal layer is always sandwiched by either two nitrogen or carbon monolayers. On the other hand, the versatility of these materials also relies on their facility to be engineered into 2D monolayers with multiple transition metals, the so-called o-MXenes (double-metal solid solutions or double-metal ordered monolayers)^14^. As an example, a double-metal ordered Ti_n_Ta_4-n_C_3_ monolayer have been already evaluated as an anode for lithium storage, showing a larger efficiency than its conventional Ti_4_C_3_ counterpart^17^. Such efficiency is related to the enhanced Li-ion transport generated by the stacking of the MXene layers^18^. Although the MXene family is already large enough, other members of the MXene family need to be mentioned, these are the i-MXenes which are characterized by having ordered or randomly distributed vacancies. These can be also synthesized from their counterpart i-MAX phases. There exist 32 i-MAX phases^19^ which will generate a variety of monolayers with random and ordered vacancies. The first i-MAX phase ((Mo_2/3_Sc_1/2_)_2_AlC) was synthesized in 2017^20^ and it was the precursor of the Mo_1.33_C monolayer which is obtained through selective etching. Such monolayer possesses ordered Sc vacancies. Another example is the Nb_1.33_C i-MXene in which the vacancies are randomly distributed, and it is obtained from the precursor i-MAX phase (Nb_2/3_Sc_1/3_)_2_AlC^21^. The randomly distributed vacancies in this monolayer are generated because in the MAX phase, the Sc and Nb atoms are forming a solid solution. In this way, the precursor i-MAX phase is key for the atomic arrangement of the resultant i-MXene^20,21^. The i-MXenes may be also as versatile as the mono-metal or the o-MXenes. They could be also engineered to form double-metal solid solution MXenes, if advantage is taken of the intrinsic vacancies in the monolayers. i-MXenes can be used in electronic and optical devices^14^ as well as in energy, catalysis, and environmental applications^19,22^.

Due to its high versatility, high surface area, and easy way of modification by adding different functional groups, MXenes point to be key in environmental remediation applications^23,24^. From these, desalination of seawater and brackish water is key. However, MXene membrane science is still in an early stage and research is needed to understand and explain the desalination processes, the device performance, and industrial scaling^25^. In this way, several research papers have appeared in which MXenes enhance the cations trapping to get efficient desalination membranes. Ti_3_C_2_T_x_ have demonstrated high selectivity to metal cations with different charges as well as effectivity larger than the one shown by graphene oxide and some other carbon-based nano materials^26^. Ti_3_C_2_T_x_ membranes were also engineered with Al^3+^ ions to prevent swelling while retaining a high salt rejection and fast water fluxes, promising scalability^27^. Ti_3_C_2_T_x_ has also been used as an intercalation material in membranes to enhance the desalination via capacitive deionization^28,29^. Also, fabricating surface charged MXene membranes generates a performance during nanofiltration and/or forward osmosis processes^30^. Research in desalination processes has been focused to Ti_3_C_2_T_x_ MXene. From an atomic perspective, ion sieving has been analyzed where ions of different charges feel different diffusion barriers throughout the membrane, where the MXene interlayer distance contracts or expands upon interacting with such ions^31^.

In a recent work, desalination via intercalation was analyzed for the Mo_1.33_CT_x_ i-MXene/Carbon nanotube electrodes, where Na^+^ and Cl^−^ are efficiently removed in the treatment of seawater and brackish water. Such process was achieved without the need of an exchange membrane, so it is hypothesized that the MXene layer is the one carrying the nanofiltration process by trapping Na^+^ and Cl^−^ at the same time. The carbon nanotubes in the electrode serve to avoid MXene re-stacking^32^. Although Mo_1.33_C MXene evidenced a high efficiency for the trapping of Na^+^ and Cl^−^ in the experimental setup, no atomic-scale understanding of such process exists in the literature.

Considering the previous discussion and the proved importance of the Mo_1.33_CT_x_ MXene in the Brackish and Seawater water desalination process via cation and anion intercalation^32^, in this work we have performed a comprehensive atomic scale density functional theory study to understand the way Na and Cl atoms get trapped on the Mo_1.33_CT_x_ monolayer (T_x_ = OH groups) and their behavior after adsorption. We explain the viability of intercalation in terms of the atomic Na and Cl electronegativities. Na^0^ atoms form bonds with the oxygen atoms of the functional groups whereas the Cl^0^ atoms prefer to interact with the hydrogen atoms of the hydroxyl groups. Once the interaction with the MXene layer happens, Na^0^ and Cl^0^ behave as Na^+^ and Cl^−^, respectively. Our study suggests that functionalizing MXenes with hydroxyl groups will make them viable for Brackish and Seawater water desalination in which both Na and Cl ions are efficiently removed.

## Method

The atomic scale understanding of the Na^+^ and Cl^−^ trapping after the brackish and sea desalination water process promoted by Mo_1.33_C(OH)_2_ was treated though density functional theory calculations, as implemented in the Vienna Ab-initio Package^33–36^ code. The one-electron wavefunctions were treated with the projector augmented wave method as derived by Kresse and Joubert^37^. The electronic states were expanded in a plane wave basis set with an optimized cutoff energy of 400 eV. Non-classical electron–electron interactions (exchange–correlation) were approximated with the generalized gradient approximation using the Perdew-Burke-Ernzerhof functional form^38^. i-MXenes are experimentally obtained with several functional groups, when hydroxyl groups are involved, long-range interactions may appear.Therefore, we considered the description of dispersion-corrected van der Waals interactions through the Grimme-D3 functional correction^39^. In this case, we considered that Na^+^ and Cl^−^ were previously separated from the water (commonly Na^+^ and Cl^−^ are dissolved into water) and would adsorb as atomic species Na^0^ and Cl^0^ on different i-MXene high symmetry sites. To reach convergence in a system, the following criteria was used: the energy must be lower than 1 × 10^−4^ eV for two consecutive electronic steps, and the norm of all atomic forces must be smaller than 0.01 eV/Å. To evaluate the electronic states at the reciprocal lattice, the Brillouin zone was sampled using an equally distributed^40^ k-points mesh with a volume of 12 × 12 × 1. To obtain the electrostatic potential isosurface and the Bader charges, the most stable adsorption models were converged with a denser k-points mesh of 24 × 24 × 1. The Bader charge analysis code^41^ was used to obtain the valence charge transfer in the stable models.

## Results and discussion

**Figure 1:**
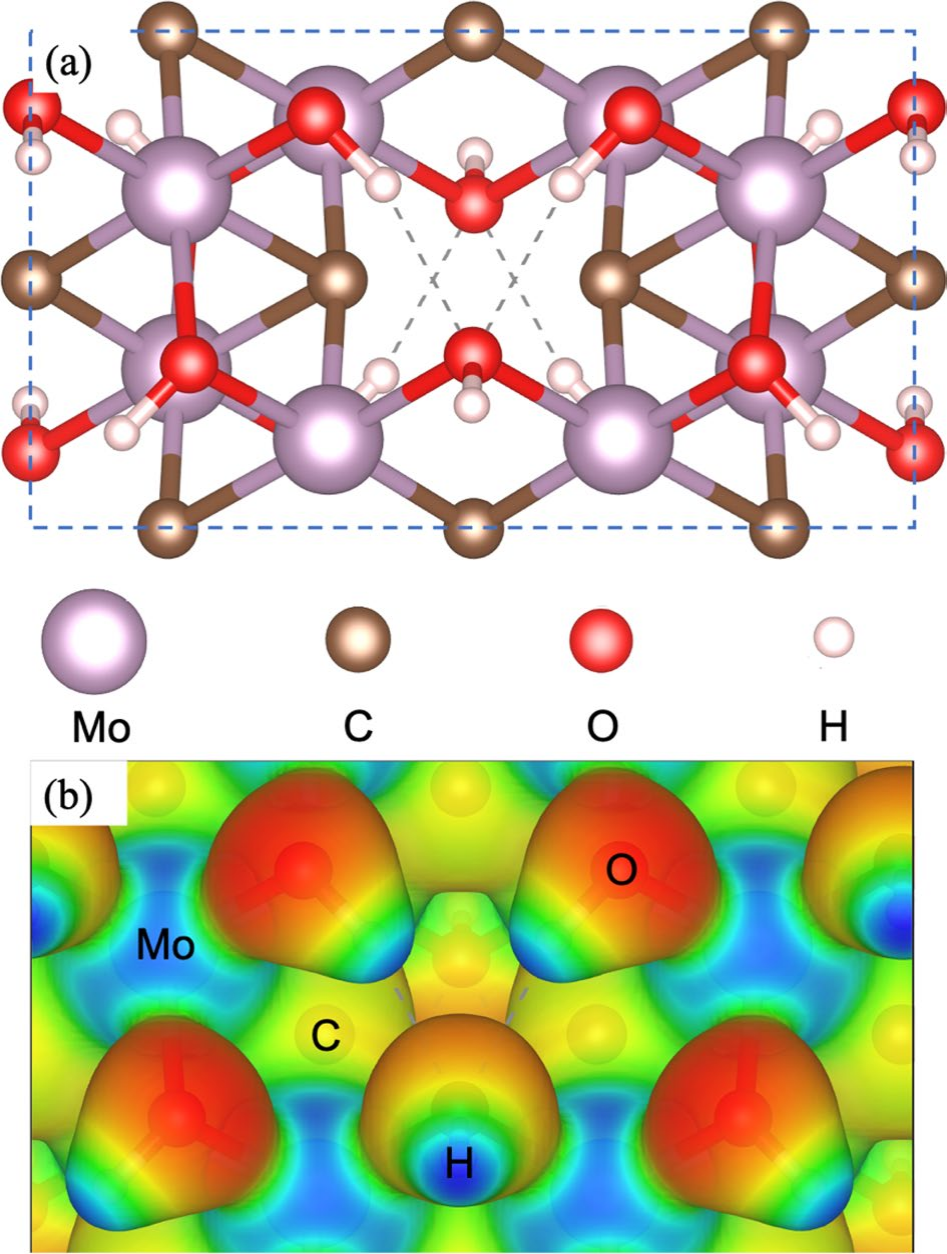
(**a**) Model system of the Mo_1.33_CT_x_ i-MXene, where T_x _(X = 2) are OH functional groups. (**b**) electrostatic potential isosurface showing the electron accumulation (red) and depletion (blue). The unit cell is highlighted as a dashed blue square.

In this section, we describe the Mo_1.33_CT_x_ monolayer and explain—at the atomic scale—the way it helps to trap Na^+^ and Cl^−^ from saline and brackish water. The Mo_1.33_C structure is typically functionalized with OH, O, and F atoms. It has been found that the Mo_1.33_CT_x_, (X=2), with T = O, is unstable^42^. However, T_x_ = OH and T_x_ = F generate stable structures which are consistent with the experimental findings. It was also shown that when there is combination of F and O as functional groups (1O:2F, 2O:1F), the system also is also stable^42^. The species observed experimentally by XPS^42^ are 50% F, 25% O and 25% OH suggesting that the O/F ratio is 50/50, but with half of the O atoms in OH form. In this work, we used the stable OH functionalized structure in which the ordered vacancies are occupied by OH functional groups (see Fig. 1a). A similar structure has been reported in previous studies^22,42^.

Once the atomic structure for the Mo_1.33_C(OH)_2_ was defined, we proceeded to calculate its electrostatic potential isosurfaces (electron accumulation is depicted in red, whereas electron depletion is depicted in blue). As expected, since the O atoms are the most electronegative, they have electron accumulation, whereas the H and Mo atoms have electron depletion (see Fig. 1b). Considering their electronegativities, O atoms would be available to bond with electropositive atoms like Na and electropositive atoms in the structure, like H, would bond to electronegative atoms like Cl. In the work by Srimuk et al.^32^, it was demonstrated experimentally that Mo_1.33_CT_x_ was able to eliminate Na^+^ and Cl^−^ from brackish and seawater with high salt concentration. However, in their work the role of the functional groups was not clarified. It was hypothesized that O or F atoms may be the ones involved in the trapping. However, considering their electronegativities—very similar to Cl atom—, they would bond to Na^0^ but not to Cl^0^ atoms^43^. Also, Srimuk et al.^32^, did not mention the OH groups, which are 25% of the coverage in the Mo_1.33_CT_x_ MXene. OH groups are more promising for Na^0^ and Cl^0^ trapping since they possess both electronegative O and electropositive H atoms. Using the information coming from the electrostatic potential isosurface, we tried several adsorption sites for both Na^0^ and Cl^0^. Adsorption sites were considered on top of the surface layer and bonded to Mo atoms. To avoid getting trapped in a local minimum, for each site, we started at different perpendicular distances. For example, site S_1_ is the same as S_1a_ but the last one is closer to the Mo layer.

**Figure 2:**
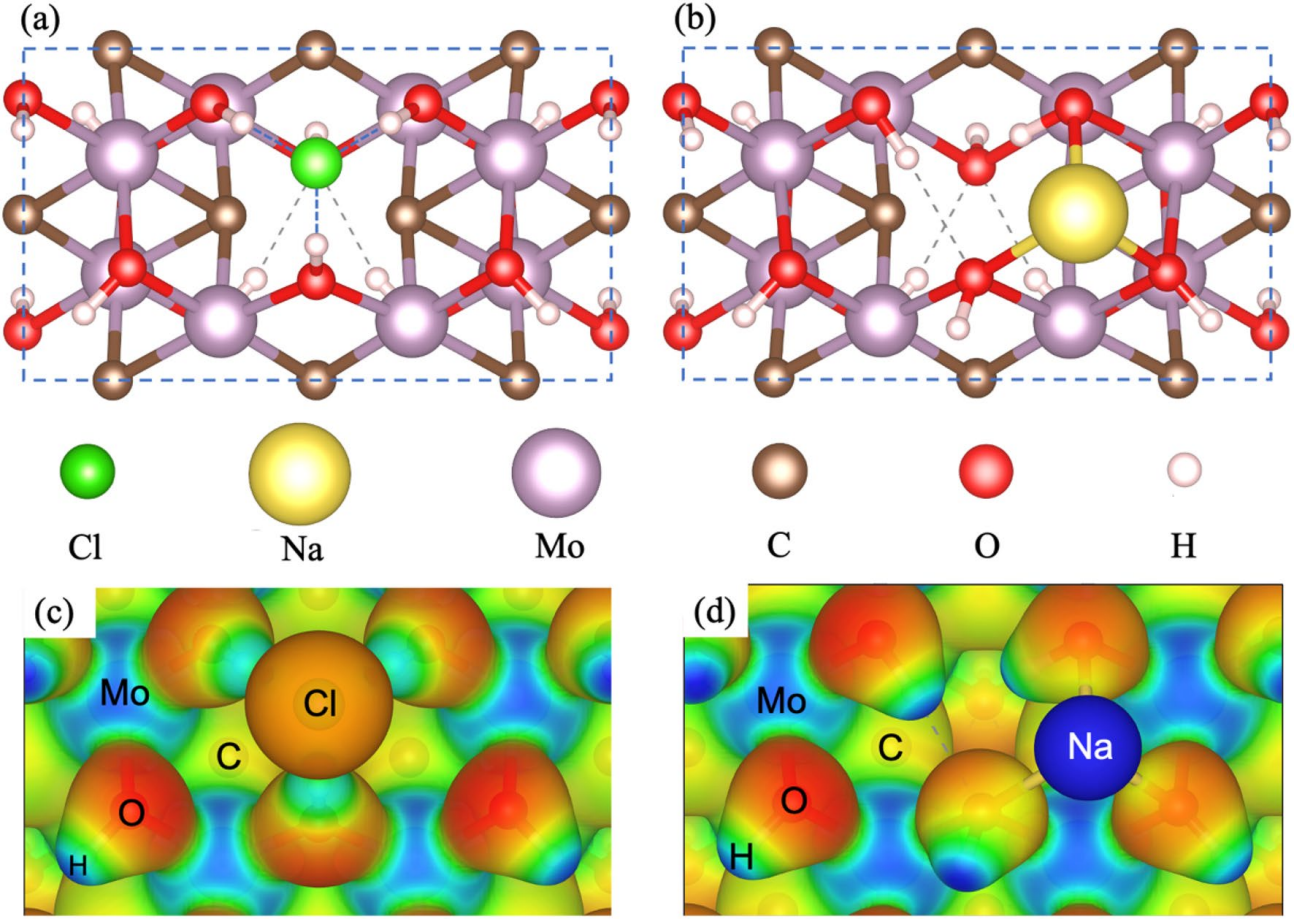
(**a**) Ball and stick models of the most stable adsorption sites (**a**) and (**b**) for Cl^0^ and Na^0^ on the Mo_1.33_C(OH)_2_. (**c**) and (**d**) electrostatic potential isosurfaces showing the electron accumulation (red) and depletion (blue) for both Cl and Na atoms interacting with the MXene. Notice that after interaction Cl^0^ reduces to Cl^−^ and Na^0^ oxidizes to Na^+^ just as when dissolved into water. Unit cell in (**a**) and (**b**) is highlighted as a dashed blue rectangle.

Upon structural optimization of all models, we obtained the adsorption energy for all stable sites, see Table 1. As a general trend, after atomic relaxation of the adatoms at different positions, we observed that all S_xa_ models transform into the S_x_ ones. Upon adsorbing Cl^0^ atoms on all defined sites (see Fig. S1), S_1_, S_5_, and S_8_ sites end up as S_7_ structures in which the Cl atom is forming three hydrogen bonds with the surface H atoms. In contrast, S_6_ stabilizes on a bridge-like site formed by two OH groups. S_4_ stabilizes as a distorted S_1_ site and is not further considered. Finally, the S_3_ site turns into the S_2_ site, which is the most stable structure. Three main adsorption sites were identified as S_7_, S_6_, and S_2_, the last being more stable than S_7_ and S_6_ by 0.09 eV and 0.26 eV, respectively. S_7_ and S_2_ are similar. In both sites, Cl forms three hydrogen bonds with the OH groups, the only difference is that S_2_ is located at the Mo vacancy site. The S_6_ site is structurally different because the Cl atom is located at a bridge-like position interacting with a couple of Hydrogen atoms. Since S_6_ and S_7_ are less stable than the S_2_ site, they are not further considered for the analysis. However, the main interaction in all observed models for Cl is through hydrogen bonds. The most stable S_2_ site is depicted in Fig. 2a. S_7_ and S_6_ adsorption sites are shown in Fig. S2.

**Table 1:** Adsorption energies (in eV) of the stable adsorption models for Cl^0^ and Na^0^ atoms on the Mo_1.33_C(OH)_2_ MXene.

Adsorption site (Cl)	Adsorption energy	Adsorption site (Na)	Adsorption energy
**S**_**2**_	− 2.04	S_2_	− 0.99
**S**_**6**_	− 1.78	S_3_	− 1.36
**S**_**7**_	− 1.95	S_4_	− 1.22
		S_5_	− 0.82
		S_6_	− 1.21

Figure 2c shows the electrostatic potential isosurface for Cl in the S_2_ site, which makes evident the depletion of charge on the hydrogen atoms and the accumulation of charge on the adsorbed Cl atoms and the O functional groups, here is evident that after adsorption, Cl^0^ is reduced to Cl^−^. Notice that there is a local charge redistribution due to the Cl^−^ presence.

With respect to Na^0^ adsorption (see Table 1), the same sites were considered (see Fig. S1). As expected, different results emerge due to the chemistry of the Na atoms. Since Na atoms are electropositive, the interaction with the monolayer is through the oxygen atoms. The most stable site is S_3_. In this case, the Na atom forms three bonds with the oxygen atoms, whereas the neighbor Hydrogen atoms reorient to preclude interaction with Na, an expected behavior since H and Na are both electropositive (see Fig. 2b). Site S_2_ is less stable than site S_3_ by 0.37 eV and experiences a slight shifting towards S_5_ (see Fig. S3). S_4_ site stabilizes in a threefold coordination with O atoms, however it is 0.14 eV less stable than the S_3_ site. The main difference is that S_4_ sits on top of a molybdenum atom (see Fig. S3) and the S_3_ site is on top of a hollow site. Upon adsorbing the Na atom on S_5_ site (Fig. S3), we noticed that it forms a double bonded configuration with the O atoms of the OH functional groups. This adsorption site is the less stable, with an energy 0.54 eV higher than S_3_. S_1_ and S_7_ are not stable, after structural relaxation evolve to S_5_. This happens since these sites try to preclude the direct H-Na interaction generated with their neighboring OH group. Finally, the S_6_ site (Fig. S3) stabilizes with the same relative energy than S_4_ (they are degenerated in energy). The main difference between S_6_ and S_4_ is that S_6_ sits nearby the Mo vacancy site—occupied by an OH group—whereas S_4_ is far from the vacancy site, see Fig. S3 for structural details.

The electrostatic potential isosurface of the most stable S_3_ site can be seen in Fig. 2d. Notice that once Na^0^ interacts with the MXene layer gets oxidized to Na^+^. Near the Na^+^, O atoms accumulate charge. On the other hand, the Na^+^ shows a strong charge depletion. The electrostatic potential isosurface of the atoms far from the adsorbates is like the one depicted in the surface without adsorbates, evidencing a local effect of the adsorbed species.

**Figure 3:**
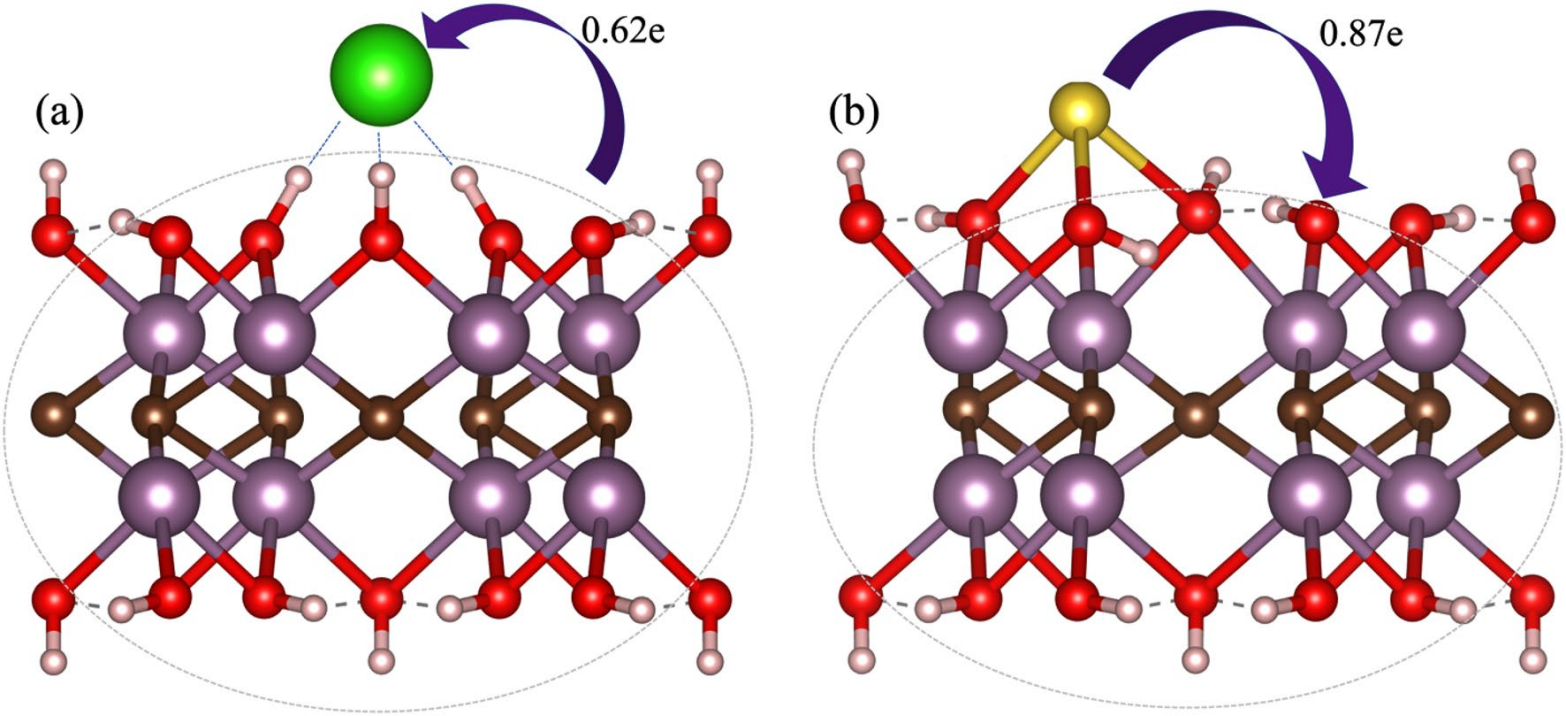
(**a**) Schematic of the charge transfer from the substrate to the Cl^−^, and (**b**) atomic model for the charge transfer from the Na^+^ to the substrate. The charge transfer was obtained through a Bader charge analysis. Violet arrows denote the charge transfer direction.

**Figure 4:**
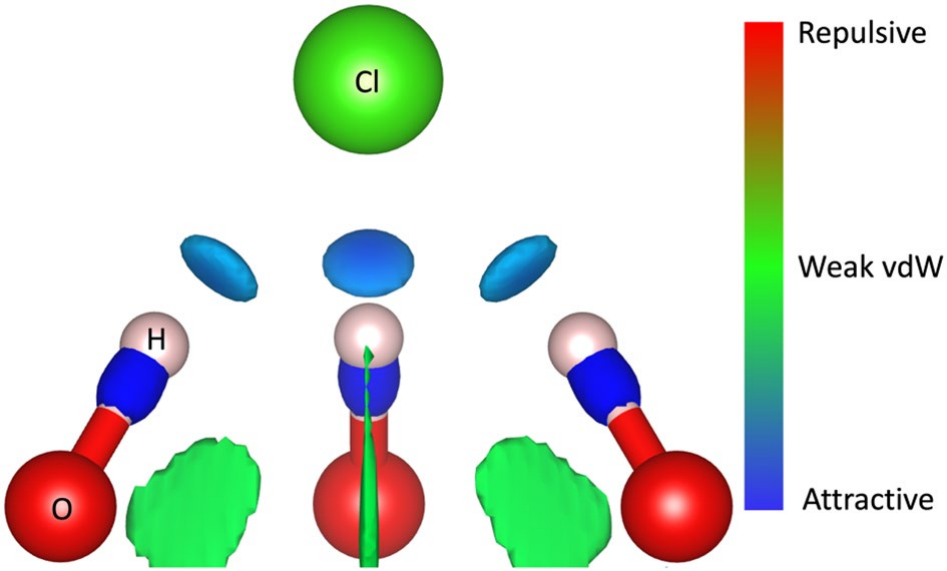
The non-covalent interaction index for the Cl^−^ adsorbed on the Mo_1.33_CT_x_ i-MXene. Gradient density isosurface was set to s = 0.5. $sign({\lambda }_{2})\rho$ is considered between − 0.05 and 0.05 to correctly image non-covalent interactions.

To gain a deeper understanding into the charge transfer between the surface and the adsorbates, we determine the Bader charges through the atoms-in-molecules topological analysis^41^. Figure 3a clearly shows that once adsorbed Cl^0^ reduces to Cl^−^, with the charge density transfer of 0.62e from the monolayer to the Cl^−^, despite their weak interaction. It is well known that hydrogen bonds are result of several energy contributions. These are: electrostatic interaction, charge transfer interactions, $\pi$-resonance assistance, cooperative effects, Pauli repulsion, dispersion interactions, and secondary electrostatic interactions^44^. In this case, one part of the hydrogen bond is the charge transfer. However, it seems that there are other contributions of electrostatic character. To prove this, we plotted the non-covalent interaction index which is based on the density and its derivatives (the so-called reduced density gradient). In principle, from density—key in DFT—one can obtain all properties of the physical systems. Then, the reduced density gradient allows to identify non-covalent interactions at low-density (low gradient) regions. They appear as spikes, each related to a different non-covalent interaction^45^. These interactions have different character, they may be attractive (hydrogen bonding) or repulsive (steric hindrance). To distinguish between them, the second eigenvalue of the electron-density matrix Hessian is used^45^. This eigenvalue takes negative values for bonding interactions, like hydrogen bond. In contrast, it takes positive values when interactions are antibonding^45^. Then, the second eigenvalue of the Hessian matrix multiplied by the density (which gives us details of the interaction strength) is a way to visualize and understand weak interactions. In Fig. 4, we plotted the non-covalent interaction index isosurfaces considering s = 0.5 in a range of $sign({\lambda }_{2})\rho$ from − 0.05 to 0.05. A red–green–blue color code is used to depict the interaction zones. Red depicts repulsive interactions, green stands for weak van der Waals interactions that could be either repulsive or attractive depending on the sign they appear on, and blue shows the strong bonding interactions. Here we can see that the Cl^−^ has a strong attractive interaction through hydrogen bonds (blue ellipsoidal isosurfaces in Fig. 4) with three hydrogen atoms from the substrate. This characteristic electrostatic character of the interaction together with the charge transfer (0.62e from the monolayer to the Cl^−^) help explain the large adsorption energy obtained for Cl adsorption (see Table 1). Green isosurfaces evidence O–O weak interactions between the functional groups.

On the other hand, for the Na case, after adsorption Na^0^ oxidizes to Na^+^, the charge density transfer is now from the adsorbate to the monolayer. The Na^+^ atom transfers 0.87e to the monolayer (see Fig. 3b). Although Na^+^ experiences a chemical interaction with the monolayer, the adsorption energy is lower than the one obtained for Cl^−^ as previously explained in the above paragraphs.

The piece of evidence here presented helps to understand the interaction mechanism that explains the way both Cl^0^ and Na^0^ are efficiently captured by the i-MXene monolayer. Also, we provide an atomic scale understanding of the Cl^−^ and Na^+^ elimination mediated by the Mo_1.33_C(OH)_2_ from brackish and seawater with high saline content, as previously reported in the literature^32^.

## Conclusions

Experimental evidence has demonstrated that Mo_1.33_CT_x_ MXene is an efficient electrode to capture Cl^−^ and Na^+^ from sea and brackish water^32^. Understanding, at an atomic scale, the chemical and physical processes that happen in these electrodes is key to tune and improve the water desalination processes. In this paper, we report on the Cl^−^ and Na^+^ trapping on the Mo_1.33_C(OH)_2_ monolayer by first-principles density functional theory calculations. Results show that after adsorption, Cl^0^ reduces to Cl^−^ due to its interaction with the H through hydrogen bonds, these bonds have two energy contributions: electrostatic and charge transfer. This fact explains its large adsorption energy observed upon adsorbing it on the i-MXene layer. On the other hand, Na^0^ oxidizes to Na^+^ once forming bonds with the O atoms. Electrostatic potential isosurfaces show that Oxygen atoms have an affinity for the electropositive Na^+^, whereas hydrogen atoms—of the hydroxyl groups—interact with the electronegative Cl^−^. Bader charge analysis and non-covalent interaction isosurfaces clarify how Cl^−^ and Na^+^ attach to the MXene layer. Na^+^ donate charge to the i-MXene, whereas for Cl^−^, the charge density goes from the substrate to the adsorbate. Our findings explain at the atomic scale how Cl^−^ and Na^+^ are efficiently removed from sea and brackish water based on their affinities with the functional groups present in the Mo_1.33_C(OH)_2_ i-MXene.

## Supplementary Information

Supplementary Figures.

## Data Availability

Authors declare that the main data supporting the findings of this study are contained within the paper. The row data is available from the corresponding author upon reasonable request.
