# Supplementary material for: Atomic-scale understanding of the Na and Cl trapping on the Mo1.33C(OH)2-MXene
Source: Sci Rep. 2022 May 18;12:8340. doi: 10.1038/s41598-022-12177-6 (PMC9117310; doi:10.1038/s41598-022-12177-6)
Supplement: Supplementary file 1 — Supplementary Figures. [file 41598_2022_12177_MOESM1_ESM.docx]

**Supplementary information**

**Atomic-scale understanding of the Na and Cl trapping on the Mo_1.33_C(OH)_2_-MXene**

**J. Guerrero-Sanchez^1^, Dalia M. Muñoz-Pizza^2,3^, Ma Guadalupe Moreno-Armenta^1^, Noboru Takeuchi^1^**

*^1^Centro de Nanociencias y Nanotecnología, Universidad Nacional Autónoma de México, km.107, Apdo. Postal 14. Carretera Tijuana-Ensenada, Ensenada, Baja California, México.*

*^2^Colegio de la Frontera Norte, Departamento de Estudios Urbanos y del Medio Ambiente, Tijuana, Baja California, Mexico*

*^3^Facultad de Ciencias Marinas, Universidad Autónoma de Baja California, Ensenada, Baja California, Mexico.*


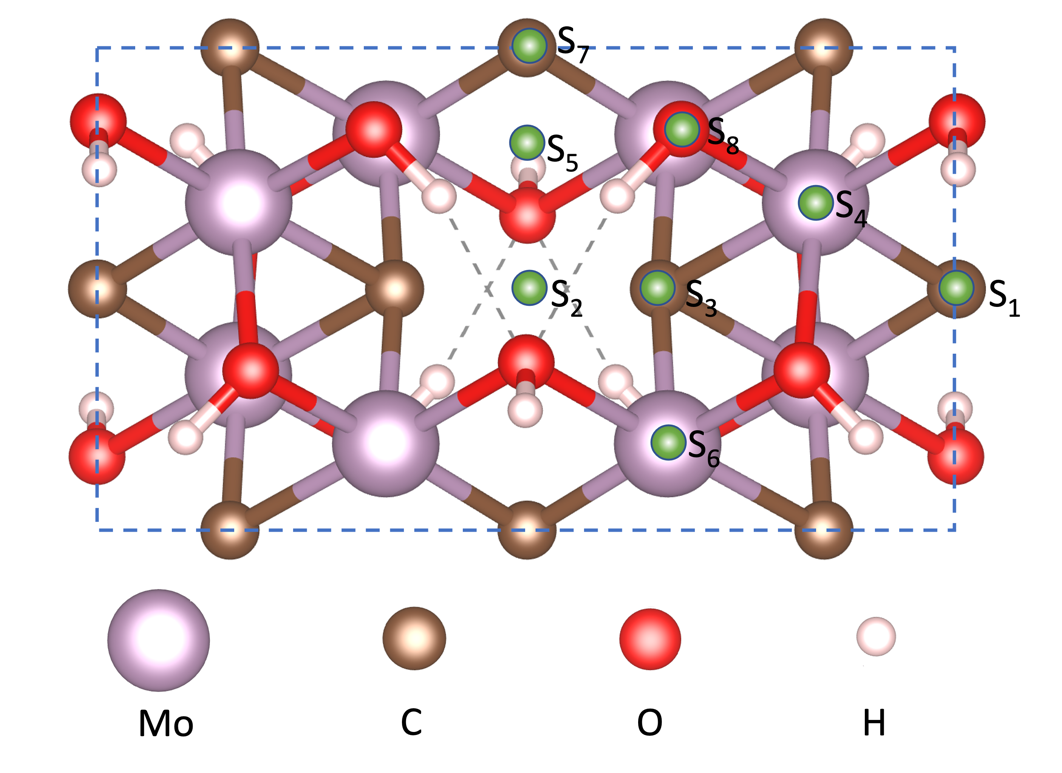


Figure S1. High-symmetry adsorption sites for the Cl^0^ and Na^0^ atoms on the Mo_1.33_C(OH)_2_ sheet.


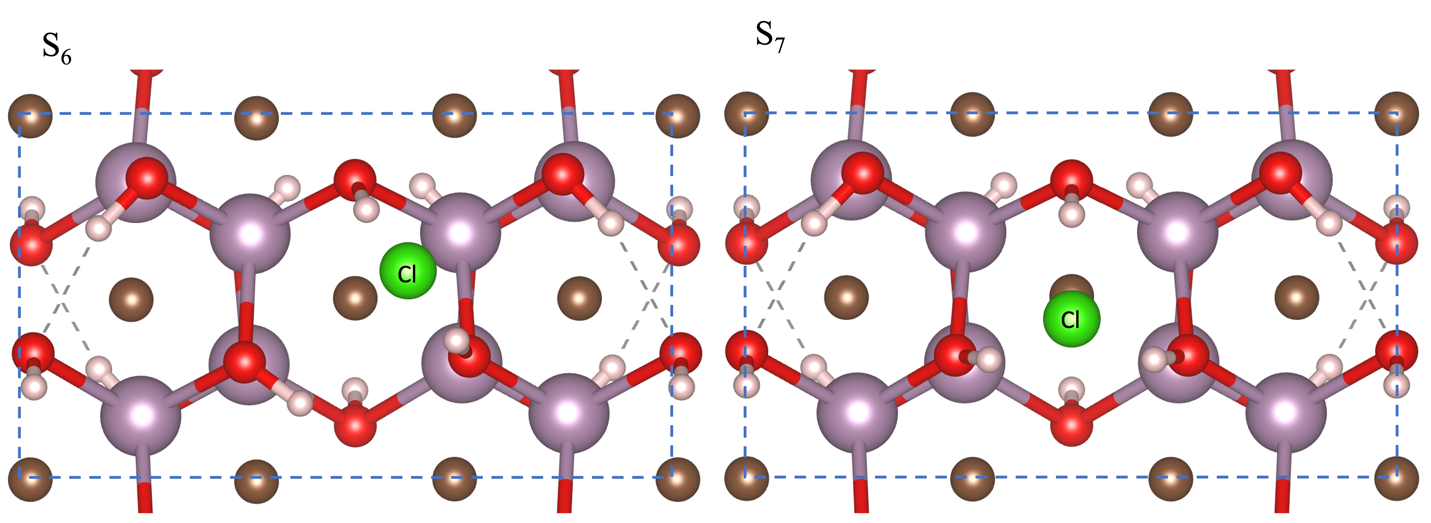


Figure S2. Ball and stick models of the stable adsorption sites for Cl^0^ atoms on the Mo_1.33_C(OH)_2_ sheet.


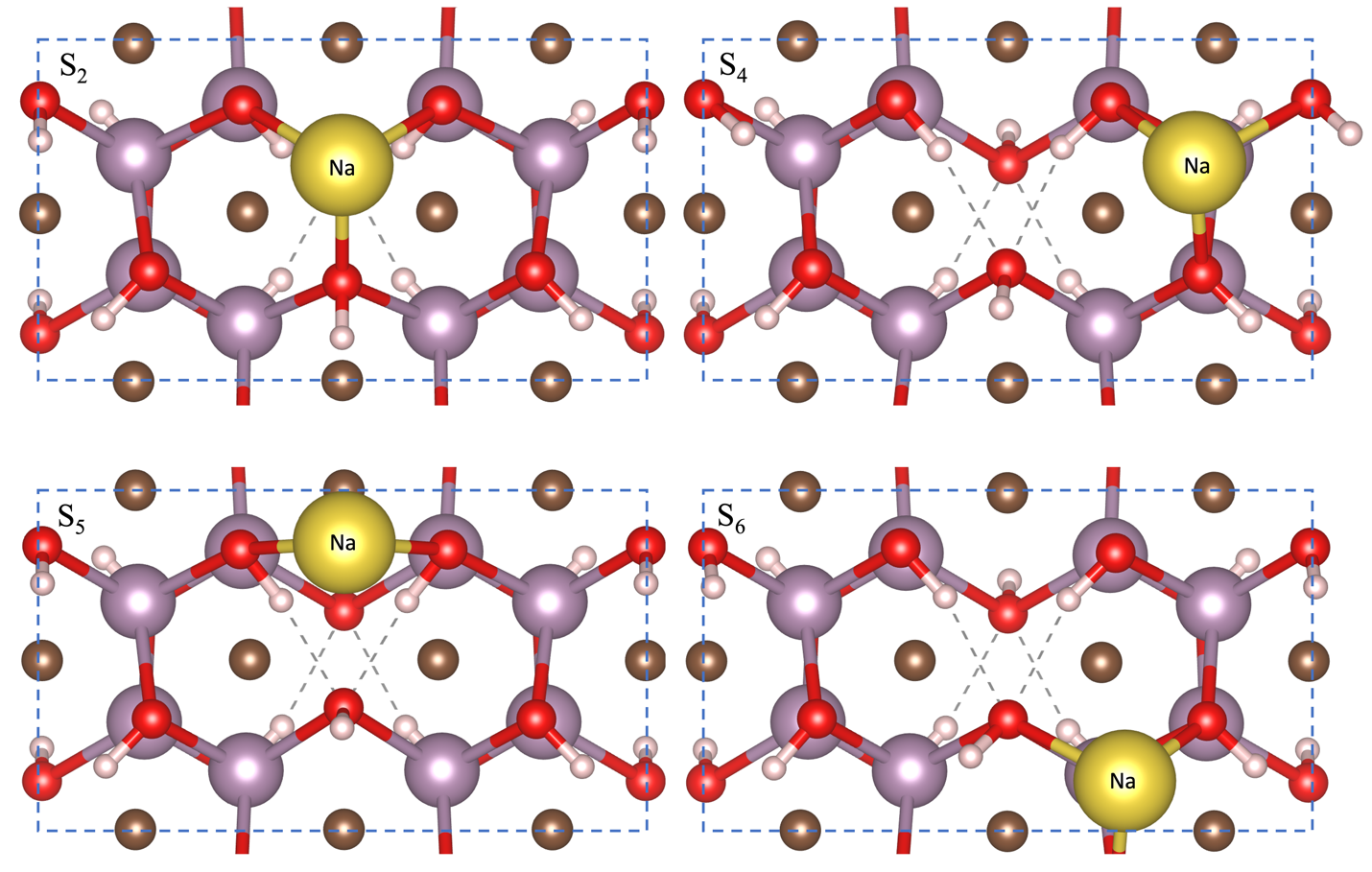


Figure S3. Ball and stick models for the stable adsorption sites of Na^0^ atoms on the Mo_1.33_C(OH)_2_ monolayer.
